# PROMETEO: Infrastructure Remote-Control and Geophysical Monitoring System of the INGV Osservatorio Vesuviano

**DOI:** 10.3390/s26185914

**Published:** 2026-09-18

**Authors:** Aldo Benincasa, Antonio Caputo, Francesco Liguoro, Giovanni Scarpato, Massimo Orazi, Roberto Manzo

**Affiliations:** Istituto Nazionale di Geofisica e Vulcanologia (INGV), Osservatorio Vesuviano, 80124 Napoli, Italy; aldo.benincasa@ingv.it (A.B.); antonio.caputo@ingv.it (A.C.); francesco.liguoro@ingv.it (F.L.); giovanni.scarpato@ingv.it (G.S.); massimo.orazi@ingv.it (M.O.)

**Keywords:** IoT monitoring, sensor networks, infrastructure monitoring

## Abstract

**Highlights:**

**What are the main findings?**

- PROMETEO integrates MQTT, SNMP, and MODBUS into a unified IoT-based supervisory platform for geophysical monitoring.
- Real-time correlation of energy, environmental, and connectivity data improves fault diagnosis and infrastructure resilience.

**What are the implications of the main findings?**

- Predictive analysis and differential diagnosis reduce unnecessary field interventions and maintenance costs, particularly for remote mobile stations.
- Operational deployment at Vesuvius, Campi Flegrei, and Ischia demonstrates the system’s applicability to monitoring networks in active volcanic areas.

**Abstract:**

The continuous operation of the geophysical monitoring networks managed by the Istituto Nazionale di Geofisica e Vulcanologia (INGV)–Osservatorio Vesuviano relies on the reliability of a highly distributed infrastructure deployed in active volcanic areas. In these contexts, failures affecting power supply, environmental control, or communication systems may lead to interruptions in data transmission and consequent loss of scientific observations. This work presents PROMETEO, an integrated remote-control and infrastructure monitoring system designed to supervise heterogeneous monitoring stations through a multiparametric sensing approach. The system combines distributed sensors and intelligent edge devices for the acquisition of electrical, environmental, and connectivity-related parameters, including battery voltage, load current, cabinet temperature, signal quality, and network reachability. Data are collected and integrated in real time through standard Internet of Things (IoT) and industrial communication protocols, namely Message Queuing Telemetry Transport (MQTT), Simple Network Management Protocol (SNMP), and MODBUS, and centralized within the open-source Home Assistant platform. This architecture enables the fusion of heterogeneous sensor measurements into a unified supervisory framework for real-time visualization, alarm generation, historical storage, and trend analysis. The results show that the multiparametric correlation of sensor data significantly improves diagnostic capability, allowing rapid discrimination between power-related anomalies and communication failures, particularly in remote mobile stations. By reducing diagnostic uncertainty and limiting unnecessary field interventions, PROMETEO enhances the operational resilience of geophysical monitoring infrastructures and supports preventive and predictive maintenance strategies. The proposed system demonstrates how a scalable multiparametric sensing architecture can strengthen the reliability and continuity of monitoring networks operating in complex environmental conditions.

## 1. Introduction

**Figure 1:**
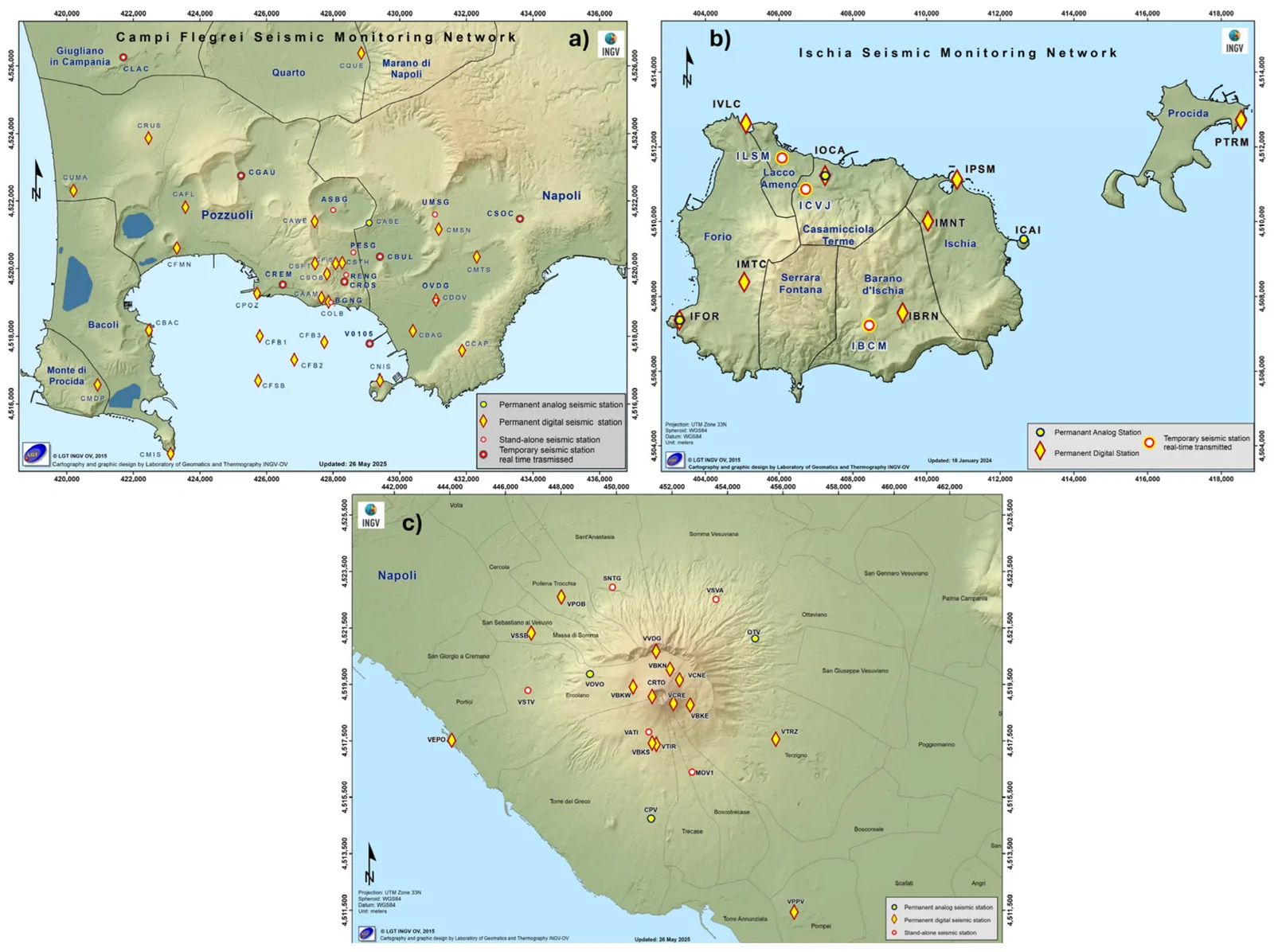
Seismic networks of the Campanian volcanic district [5]. (**a**) Campi Flegrei seismic network; (**b**) Ischia island seismic network; (**c**) Mount Vesuvius seismic network.

The monitoring networks managed by the Istituto Nazionale di Geofisica e Vulcanologia (INGV)—Osservatorio Vesuviano represent a critical technological infrastructure for the continuous observation of geophysical and environmental phenomena affecting the active volcanic areas of Mount Vesuvius, Campi Flegrei, and Ischia Island (Figure 1). These networks are characterized by a high degree of heterogeneity from both instrumental and infrastructural perspectives and include permanent and mobile monitoring stations, multiparametric sensors, data acquisition systems, communication devices, autonomous power supply units, and data processing centers. Detailed descriptions of the seismic networks at Mount Vesuvius, Ischia Island, and Campi Flegrei are provided in [1,2,3,4], respectively.

The proper functioning of these infrastructures does not depend exclusively on the quality of the acquired scientific data, but increasingly on the ability to ensure operational continuity of the stations, timely identification of infrastructure anomalies, and the possibility of targeted interventions before faults or functional degradation compromise data transmission. The environmental conditions in which many stations operate, often in remote or difficult-to-access areas, make field maintenance complex and costly; without a reliable telecontrol system, malfunctions may remain undetected for long periods, resulting in data loss and reduced monitoring effectiveness.

The PROMETEO system was developed to address these operational needs as an integrated telecontrol and infrastructure monitoring platform designed to provide a centralized, coherent, and continuously updated view of the operational status of the monitoring networks of the Osservatorio Vesuviano.

The main objective of the system is to collect, integrate, and visualize in real time diagnostic and operational information from heterogeneous components, correlating infrastructure and environmental parameters in order to identify degradation patterns before they turn into operational failures.

PROMETEO also supports preventive and predictive maintenance strategies through the continuous archiving of diagnostic parameters and their temporal analysis, reducing unnecessary interventions and optimizing the use of technical resources. From an operational perspective, the system is conceived as a direct support tool for monitoring room activities, increasing operators’ situational awareness and improving response capability to unexpected events.

Multiparametric monitoring represents a central element in the operational management of distributed monitoring infrastructures, particularly in complex and geographically extended contexts such as those managed by the Osservatorio Vesuviano [6,7,8,9]. Unlike isolated monitoring of individual components, this approach makes it possible to observe and correlate multiple operational quantities simultaneously, providing a comprehensive view of the health status of stations and of the network as a whole.

Within the operational context of PROMETEO, parameters related to electrical power supply and network connectivity play a priority role, as they represent the main limiting factors for station operational continuity. Energy monitoring makes it possible to track battery status, voltage levels, charge–discharge cycles, and the energy sustainability of autonomous stations, allowing early identification of degradation conditions and prevention of unexpected shutdowns.

Likewise, network connectivity monitoring makes it possible to assess station reachability and data transmission continuity. The integrated analysis of energy and network parameters allows rapid discrimination between power-supply problems and communication-infrastructure issues, reducing diagnostic time and improving the effectiveness of operational interventions.

The continuous archiving of energy and network parameters supports long-term trend analysis, a key element for maintenance optimization and a reduction in unnecessary field visits, especially for mobile stations or stations installed in difficult-to-access areas [3].

## 2. Materials and Methods

The PROMETEO system was designed according to a layered architecture aimed at separating the acquisition of infrastructure parameters, their transmission, and their centralized supervision. The hardware architecture is based on distributed sensors and control modules that act as intelligent edge nodes, responsible for digitizing critical parameters such as battery voltage, current, internal temperature, and network reachability. This organization allows heterogeneous diagnostic information to be collected from distributed monitoring stations and integrated into a single operational framework.

The SNMP is used within PROMETEO to monitor network devices connected to the station infrastructure, particularly routers, managed switches, and MikroTik wireless radio equipment (MikroTik SIA, Riga, Latvia). The devices act as SNMP agents and expose operational parameters through standardized or vendor-specific Object Identifiers (OIDs). PROMETEO periodically queries these OIDs at 5 min intervals to retrieve information such as device reachability, interface status, received signal strength indicator (RSSI), transmission bit rate, link quality, traffic counters, and operating time. The acquired values are converted into logical entities within Home Assistant, where they are displayed on dedicated dashboards, stored for historical analysis, and evaluated against predefined thresholds for alarm generation. This integration enables communication-link performance to be correlated with electrical and operational parameters acquired through MQTT [10] and MODBUS, thereby helping operators distinguish network degradation from power-supply or equipment failures.

### 2.1. System Architecture

**Figure 2:**
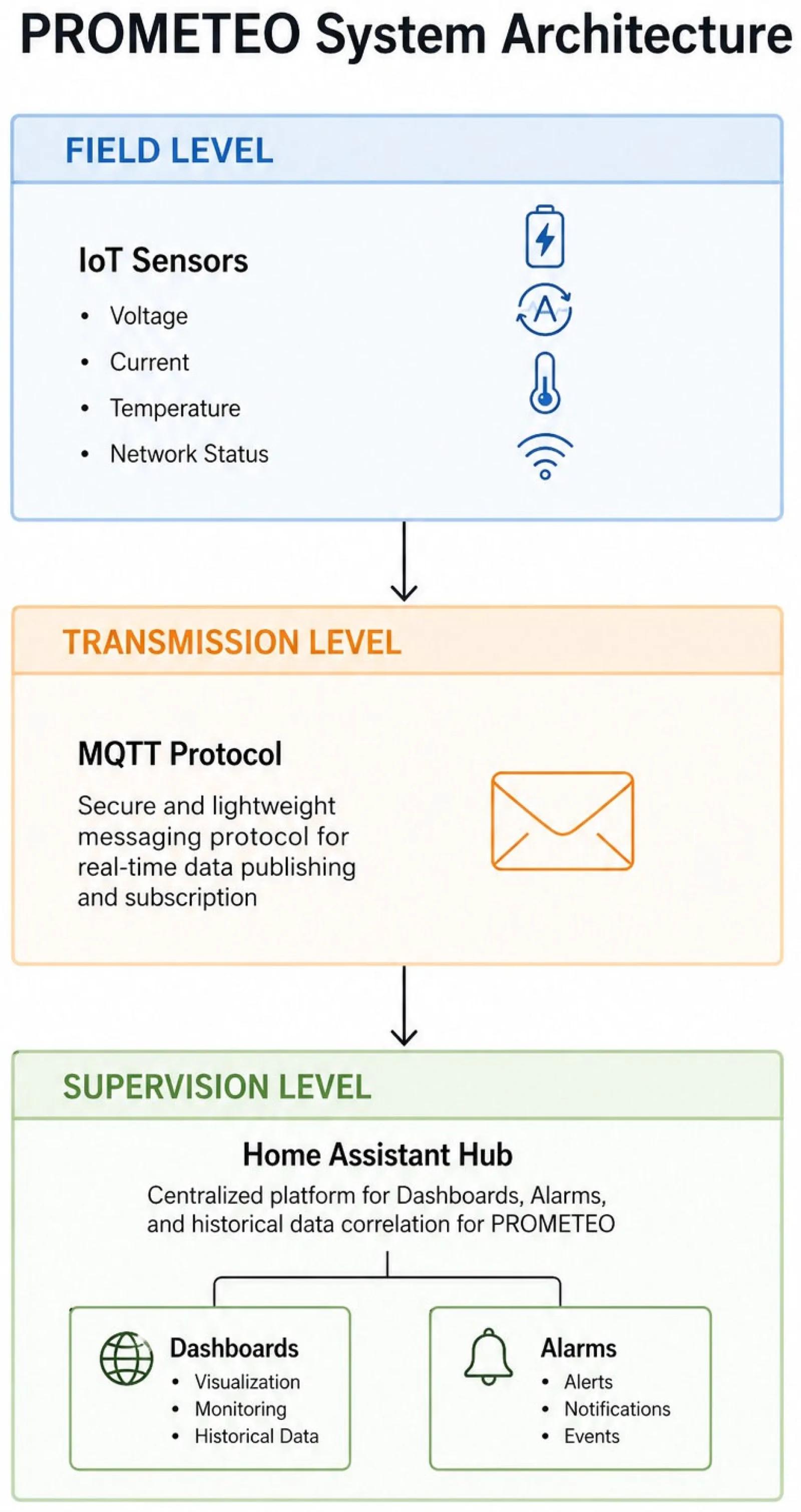
Conceptual architecture of the PROMETEO system.

The conceptual architecture of the PROMETEO system is summarized in Figure 2. The diagram provides an overview of the main functional levels of the system and of the logical flow of information from the monitoring stations to the supervisory environment. At the lower level, the field infrastructure generates diagnostic data describing the operational status of the stations, including electrical, environmental, and connectivity-related quantities. These parameters are then transferred through a communication layer toward a central supervision level, where they can be visualized, stored, and used to support alarm generation.

Figure 2 therefore represents the general methodological scheme adopted in PROMETEO: infrastructure parameters are continuously acquired in the field, transmitted in near real time, and made available to operators through centralized interfaces. This layered representation highlights the separation between sensing, communication, and supervision functions, which is a key requirement for ensuring scalability and adaptability to both permanent and mobile monitoring stations deployed in heterogeneous environments.

Within this framework, the architecture is not limited to the acquisition of individual diagnostic variables but is designed to support the correlation of multiple operational parameters. This multiparametric approach enables a more comprehensive interpretation of station conditions and facilitates the identification of different classes of anomalies, such as power-supply degradation, environmental stress within equipment enclosures, or temporary loss of network connectivity. The detailed implementation of the system architecture and the adopted technological components are described in the following subsection.

Within the multiparametric networks of the Osservatorio Vesuviano, the Internet of Things (IoT) represents the fundamental layer for continuous monitoring of the operational status of the stations [11,12]. The hardware architecture is based on distributed sensors and control modules that acquire critical parameters such as power-supply status, battery voltage and current, cabinet internal temperature, network reachability, transmission activity, and local storage usage. These devices operate as intelligent edge nodes capable of digitizing the physical status of the infrastructure and making it available for central processing, according to the paradigms described in the literature for IoT architectures applied to distributed monitoring [1,2,3,4].

The core of the PROMETEO system is Home Assistant, an open-source platform used as the central supervisory hub. The choice of Home Assistant is motivated by its modular architecture, which enables the integration of a large number of heterogeneous devices and protocols, a feature that is essential in complex scientific and infrastructural contexts.

Within PROMETEO, Home Assistant receives diagnostic data from field devices and converts them into logical entities that can be visualized through customized dashboards for monitoring room operators and used as triggers for intelligent automation and alarm management.

Other software monitoring systems, such as openHAB, Hubitat, and Domoticz, perform similar software-hub functions by collecting heterogeneous data and organizing the information within their own databases [13,14]. Home Assistant, openHAB, and Domoticz are open-source platforms, whereas Hubitat requires dedicated proprietary hardware. Among these solutions, Home Assistant is the most widely adopted and is supported by a very large community of developers, both for the platform itself and for third-party software and integrations that can subsequently be incorporated into the Home Assistant environment [13,14,15].

The main characteristics of these systems are summarized in the following comparative Table 1 [15].

The main advantages of the proposed Home Assistant–based system are its open-source nature, broad compatibility with heterogeneous devices, and flexibility in integrating custom software and sensors. Moreover, its large and active developer community ensures continuous updates, extensive support, and long-term system scalability.

**Figure 3:**
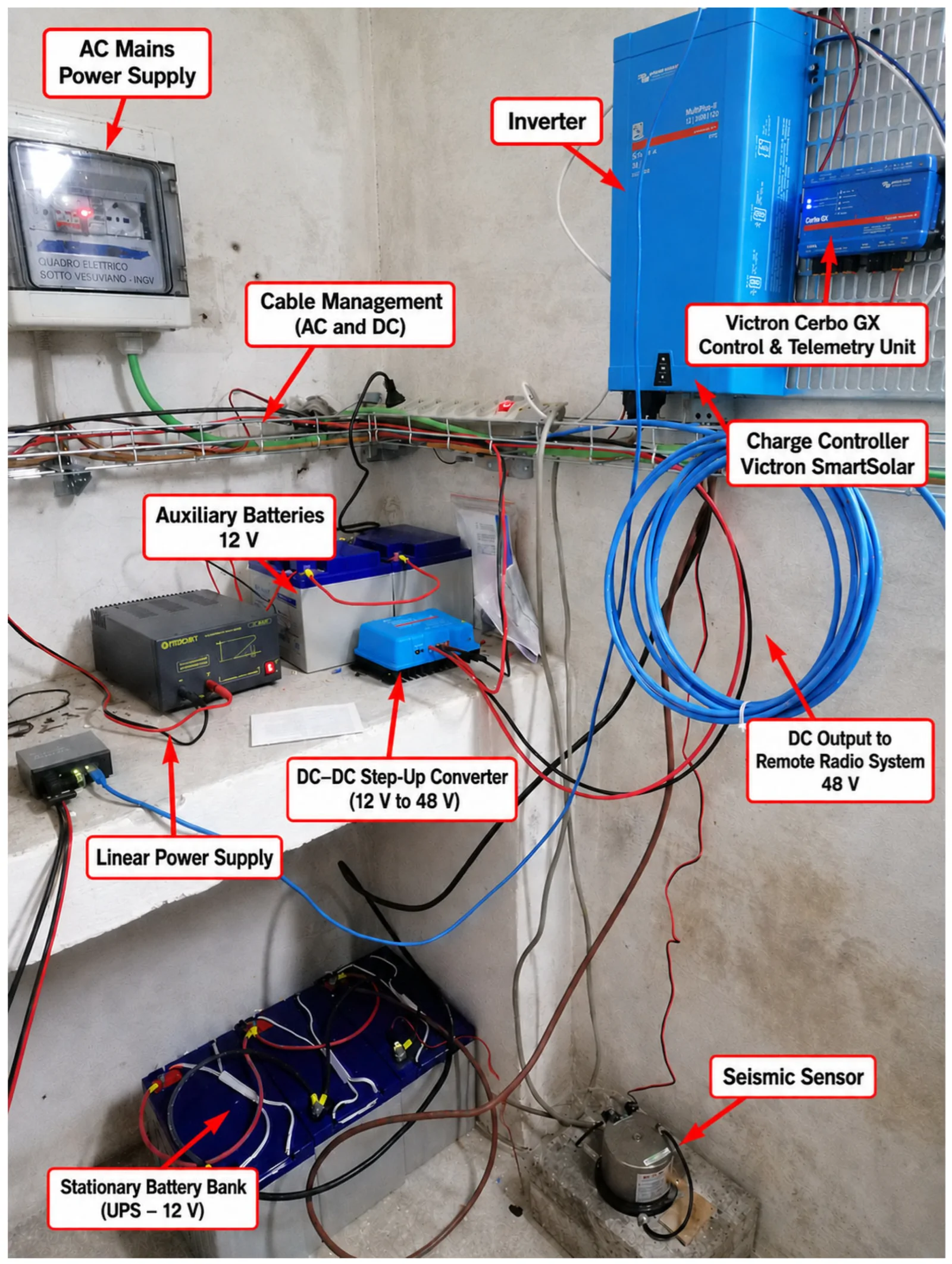
Example of a multiparametric station (CBAC site).

Figure 3 shows a representative multiparametric monitoring station installed at the CBAC site, located within a former Roman cistern inside the archaeological area of the Castle of Baia. The installation exemplifies the standard architecture of the power supply and remote supervisory system adopted within the geophysical monitoring network. The upper-right section of the station houses the inverter unit, whereas the lower compartment contains the associated stationary battery bank. In addition, the system incorporates a dedicated linear power supply exclusively intended for the operation of an analog tiltmeter, thereby ensuring enhanced electrical stability, low-noise performance, and uninterrupted functionality for this particularly sensitive instrument. Overall, the installation operates as an Uninterruptible Power Supply (UPS) system specifically designed for long-term geophysical monitoring applications. A distinctive feature of the configuration is its modular autonomy, which can be extended according to operational requirements through the integration of additional battery units. The entire infrastructure is fully remotely supervised and enables continuous real-time monitoring of several electrical and operational parameters, including residual battery capacity, mains power availability, and fault conditions affecting both alternating-current (AC) and direct-current (DC) subsystems. The upper section of the station also accommodates the charge controller together with the Victron Cerbo GX control and telemetry unit. A DC–DC step-up converter is mounted on the lateral shelf and is employed to supply power to a remote radio-transmission system. More specifically, the converter raises the DC supply voltage from 12 V to 48 V in order to satisfy the operational requirements of the communication apparatus. Two additional auxiliary batteries, together with their corresponding linear battery charger, are positioned on the same lateral shelf, while the three primary batteries devoted to the stationary power supply are installed in the lower section of the structure. The seismic sensor associated with the monitoring station is positioned behind the electrical instrumentation and mounted on a dedicated reinforced-concrete foundation specifically engineered to ensure mechanical stability, minimize environmental disturbances, and guarantee high-quality seismic signal acquisition.

The inverter shown in Figure 3 has a nominal power rating of 1200 VA. Its capacity is intentionally dimensioned not only for the instrumentation currently installed at the site but also to accommodate additional monitoring equipment that may be installed during subsequent extensions of the station.

The primary power source depends on the characteristics and location of each installation. The monitoring infrastructure can use the national electrical grid, where available, solar power, where environmental and installation conditions permit its use, and battery-based power supplies, when required. These solutions are adopted according to the specific requirements of both permanent and mobile monitoring stations.

Power-supply redundancy is implemented at selected strategic and critical sites. In these configurations, switching between different power sources is performed automatically by dedicated hardware installed at the station, rather than being commanded by PROMETEO. PROMETEO monitors the resulting failover event, records it, and provides the corresponding notification to the operators. Communication redundancy is implemented using multi-carrier and/or multichannel VPN routers of the Viprinet family (Viprinet Technologies GmbH, Bingen am Rhein, Germany), which automatically route data through the communication carriers that are available online, including LTE, Starlink, ADSL, and other available communication channels. Connectivity is therefore heterogeneous and can be implemented through LTE, Ethernet, ADSL, satellite connectivity, or other available transmission systems.

The choice of a customized discrete UPS solution is mainly motivated by the need to predetermine and control the actual operating autonomy of the monitoring station in the event of a failure of the primary power supply. Although the power consumption of the installed geophysical instrumentation is relatively low, knowing both the actual electrical load of the equipment and the exact storage capacity of the installed battery bank makes it possible to dimension the backup system according to the required operating autonomy.

**Figure 4:**
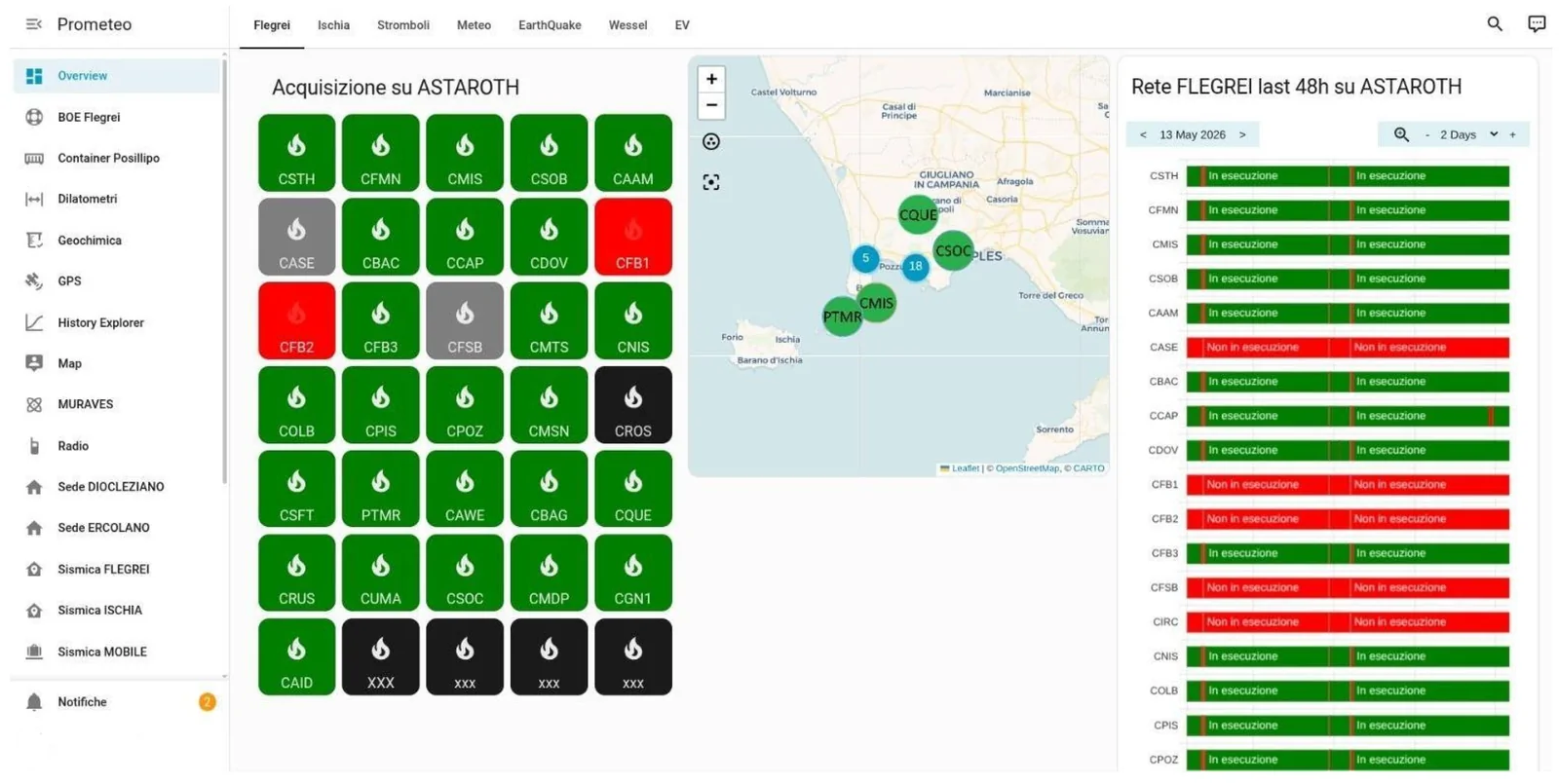
Main dashboard of the PROMETEO system for real-time supervision of the Campi Flegrei monitoring network. The left panel shows the current status of individual stations: green indicates normal operation and active data transmission; red indicates interrupted data flow; grey indicates a reachable station that is not transmitting monitoring data; and black indicates a device not detected by PROMETEO. The flame-shaped symbol identifies each monitored station. The central panel displays the georeferenced distribution of the stations, while the right panel shows their status history over the selected 48 h interval, with green segments indicating normal acquisition and red segments indicating interruptions or unavailable data.

Figure 4 shows the main dashboard of the Prometeo system, a platform designed for the remote monitoring and operational supervision of the instrumental networks deployed within the Campi Flegrei caldera. The interface provides a real-time overview of the operational status of both monitoring stations and associated sensors acquired by the system. Green panels denote stations that are correctly operating and actively transmitting data to the PROMETEO platform. Red panels indicate stations for which the expected data flow is interrupted. Grey panels identify stations that remain reachable through the communication infrastructure but are not currently transmitting monitoring data, whereas black panels indicate devices that are not detected by the PROMETEO platform. At the center of the dashboard, a georeferenced map displays the spatial distribution of the monitoring stations across the volcanic area. Stations experiencing operational issues are highlighted in red, thereby enabling the immediate identification of critical conditions and facilitating rapid diagnostic assessment by system operators. The panel located on the right-hand side of the interface presents a configurable temporal history of the network status; in the example shown, the selected observation window spans 48 h. This section allows operators to evaluate the persistence and temporal evolution of malfunctions or communication outages, thus providing essential information for both technical troubleshooting and network reliability assessment.

The left panel provides an instantaneous overview of the stations belonging to the selected monitoring network, whereas the right panel summarizes the operational status over the selected time interval. Consequently, the status displayed in the two panels may differ because the right panel reflects the temporal evolution of data transmission, while the left panel represents the current state of the selected stations.

**Figure 5:**
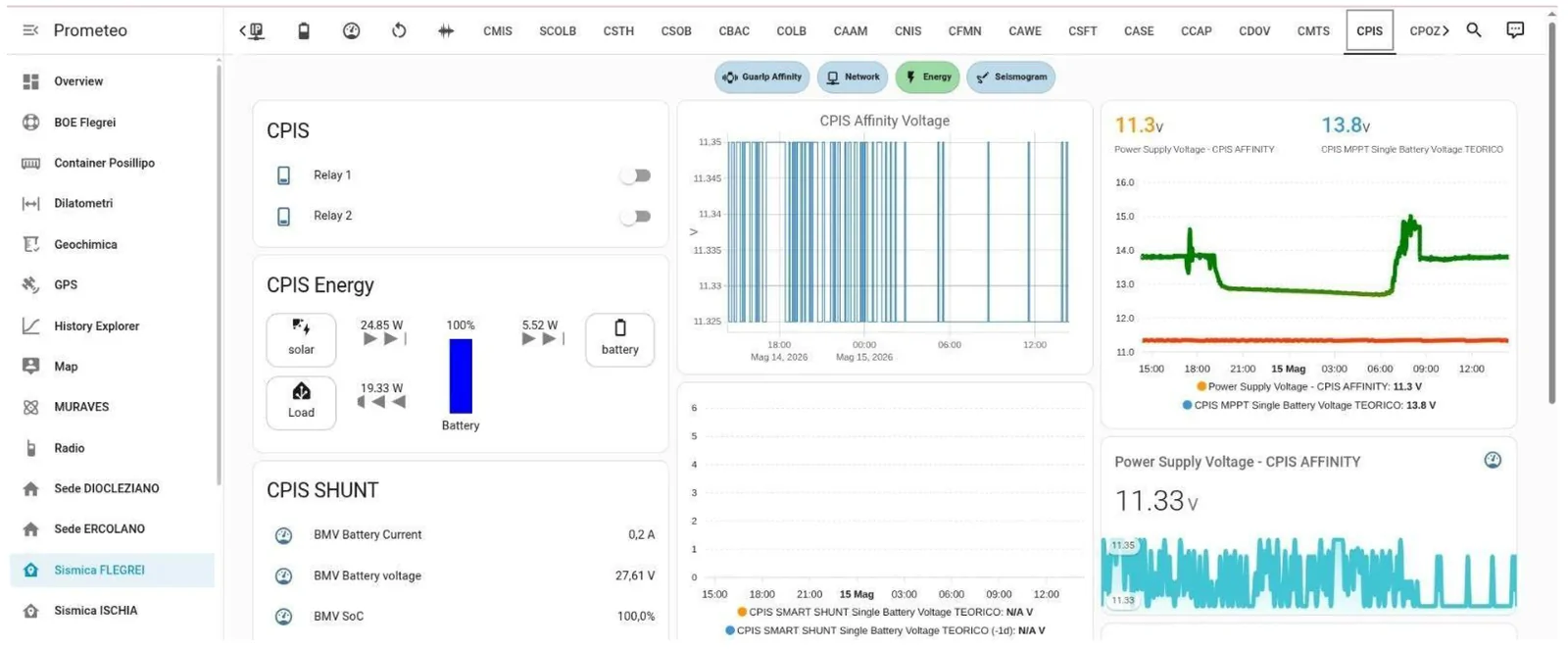
Detailed PROMETEO dashboard for the CPIS monitoring station. The upper-left panel displays the states of the remotely controlled Relay 1 and Relay 2 switches. The “CPIS Energy” panel shows the energy flow among the photovoltaic source, the electrical load, and the battery-storage system: the solar-panel, “Load”, and battery icons identify the corresponding components, while the arrowheads indicate the direction of energy flow. The thick blue vertical bar represents the battery state of charge. The thin blue vertical bars in the central graph represent variations in the monitored Güralp Affinity supply voltage over time. The blue and yellow markers shown in the graph legends identify the corresponding voltage data series and do not represent independent dashboard symbols. The lower-left panel reports battery current, voltage, and state of charge measured by the battery-monitoring system.

Figure 5 illustrates the detailed interface associated with a specific station of the seismic monitoring network, identified by the code CPIS and located within the Solfatara area of the Campi Flegrei caldera (see Figure 1). The interface is composed of multiple informational panels, each dedicated to a specific sensor or instrumental subsystem installed at the monitoring site. Each panel reports diagnostic parameters and operational metadata related to the corresponding device, thereby enabling operators to assess in real time the operational status of the station, the performance and reliability of the sensors, and the integrity of the acquired data streams. The dashboard therefore provides a comprehensive and high-resolution overview of the station’s operating conditions, constituting an essential tool for the supervision, maintenance, and reliability management of the entire monitoring infrastructure.

The left-side navigation panel provides access to the different monitoring networks, organized according to instrumental category. These include: the buoy-based monitoring network deployed in the Campi Flegrei area; the Posillipo container system dedicated to radio transmission infrastructure; the dilatometric monitoring network; the geochemical monitoring network; the GPS geodetic network; additional multiparametric monitoring networks integrated within the supervisory framework.

### 2.2. Integrated Protocols

The PROMETEO system is designed to operate in heterogeneous infrastructural contexts, typical of the volcanic areas monitored by the Osservatorio Vesuviano, where IoT devices [12], network equipment, and measurement instrumentation coexist. To guarantee interoperability, reliability, and operational continuity, the system integrates different communication protocols, each adopted according to the architectural layer and the type of data to be acquired. The following subsections describe the integration of the Message Queuing Telemetry Transport (MQTT) [10], MODBUS [16], and Simple Network Management Protocol (SNMP) [17] protocols within the supervisory platform. To guarantee efficient communication with low overhead, data produced by field devices and collection scripts are published through the MQTT protocol. MQTT is used as the main transport mechanism for operational and diagnostic data thanks to its publish–subscribe model and reduced bandwidth usage, characteristics that are particularly relevant in remote contexts or where connectivity is not continuous [10]. Within the PROMETEO system, field devices and acquisition scripts publish data in JSON format, allowing structured and easily extensible representation of information. MQTT topics are organized hierarchically so as to represent the geographical and functional context of the monitoring stations. An example of a topic used in the system is the following: ingv/vesuvio/mobile_station_01/status. The topic structure makes it possible to: distinguish networks, subnetworks, and monitoring stations; filter messages by geographic area or station type; and simplify data mapping within the supervisory platform.

Supplementary Material File S1 (“MQTT payload”) contains an example of the JavaScript Object Notation (JSON)-formatted MQTT message adopted for station telemetry transmission. Within the Home Assistant platform, MQTT data are converted into logical entities through the configuration of dedicated sensors, enabling real-time visualization, automatic historical storage, and the use of parameters for automation and trend analysis. The MODBUS TCP protocol [16] is used in the PROMETEO system for the direct acquisition of electrical and industrial quantities from field instrumentation, such as energy meters and multifunction measuring devices. MODBUS enables access to numerical registers with high update frequency, making it particularly suitable for monitoring energy and load parameters. MODBUS devices are queried via Transmission Control Protocol/Internet Protocol (TCP/IP) connection and the acquired registers are subsequently processed and scaled within the supervisory platform, separating the acquisition of raw data from their physical interpretation.

Supplementary Material File S2 (“Electrical meter configuration”) reports an example of the YAML-based configuration used for remote acquisition of electrical parameters through the Modbus/TCP protocol. The integration of MODBUS data with those acquired through MQTT and SNMP provides a unified view of the energy, operational, and infrastructural status of the monitoring stations.

Home Assistant operates as a software hub that centralizes information acquired from different systems accessible through the network by using standard communication protocols, including MQTT, MODBUS, and SNMP. The protocol actually used depends on the characteristics and communication capabilities of each device integrated into the monitoring infrastructure. IT and networking devices can be monitored through SNMP; MQTT is particularly suitable for IoT devices and energy-management modules; and MODBUS is used for commercial electrical energy meters and other industrial instrumentation. The use of MQTT, MODBUS, and SNMP within PROMETEO is therefore complementary rather than redundant, because a single communication protocol would not provide access to all the heterogeneous devices deployed within the monitoring infrastructure.

SNMP is used within PROMETEO for the monitoring of network devices connected through Ethernet infrastructures, including routers, managed switches, computers, printers, and other SNMP-enabled equipment. This allows the platform to remotely retrieve networking and operational information from these devices and integrate it with the other infrastructure parameters monitored by PROMETEO [17].

## 3. Results

The PROMETEO system has been designed to operate across the institution’s infrastructures, covering highly complex operational scenarios in the active volcanic areas of Mount Vesuvius, Campi Flegrei, and the island of Ischia. The flexible architecture of the system enables the simultaneous management of both permanent networks and the Mobile Seismic Network of the Osservatorio Vesuviano. The platform has been in routine operational use for approximately one and a half years. Table 2 summarizes the main device classes currently integrated into the system, together with their models, monitored parameters, communication protocols, and polling intervals.

PROMETEO currently supervises more than 250 monitoring stations distributed across the three volcanic areas monitored by the INGV–Osservatorio Vesuviano, in addition to the acquisition centers. On average, approximately four devices are monitored at each station. A typical configuration includes a Shelly Pro 1PM device (Shelly Europe Ltd., Sofia, Bulgaria) for monitoring the availability of the electrical power supply, a Güralp Affinity acquisition system (Güralp Systems Ltd., Reading, UK) for seismic signal acquisition, a Cerbo GX control and telemetry unit (Victron Energy B.V., Almere, The Netherlands) for power and solar-energy management, and MikroTik Wi-Fi radio equipment (MikroTik SIA, Riga, Latvia) for digital communications. The system generates several tens of operational messages per day, associated with events such as mains power availability or failure, loss of IP reachability detected through ping checks, variations in monitored parameters beyond predefined thresholds, and failover events involving network or electrical devices. No fixed maximum number of devices per station or maximum number of nodes has been defined for the current configuration; scalability depends primarily on the hardware resources allocated to the Linux server hosting the Home Assistant hub (version Linux Home Assistant OS, kernel 6.18.39-haos).

Before the implementation of the proposed remote monitoring approach, the monitoring sites were mainly managed through local inspection and diagnosis performed directly in the field by technical personnel using dedicated diagnostic instruments, such as multimeters, oscilloscopes, and network analyzers. With PROMETEO, relevant physical and operational parameters can be remotely acquired and analyzed, allowing technical personnel to identify and validate faults or anomalous conditions before reaching the monitoring site and consequently to plan specific and targeted maintenance interventions.

PROMETEO does not provide direct real-time access to the geophysical data streams. However, it provides access to JPG image frames of the seismograms, updated every 5 min, for a rapid visual overview of the recorded seismic signals. At present, the system does not perform a direct assessment of seismic or other geophysical data quality. Nevertheless, the seismometer mass position is monitored through direct access to the digitizers, and the number/status of visible GPS satellites is also acquired. Instrument calibration status and seismic noise levels are not currently monitored. A typical mobile station consists of a Lunitek digitizer ATLAS (Lunitek S.r.l., Sarzana, Italy) for local acquisition and real-time transmission; a Lennartz 3D Lite seismic sensor (Lennartz electronic GmbH, Tübingen, Germany); an LTE router manufactured by Teltonika Networks (Teltonika Rut 950) (Kaunas, Lithuania) or an LR77 LTE router (Advantech Czech s.r.o., Ústí nad Orlicí, Czech Republic) for real-time data transmission; a battery-based power-supply system equipped with an HSB115 battery charger (Microset S.r.l., Italy); and an IP65 enclosure protecting the instrumentation. The typical total current consumption is approximately 500 mA at 12 V, corresponding to an average power consumption of approximately 6 W.

The application to mobile stations represents the most critical scenario. These installations are often located in remote or difficult-to-access areas and operate under autonomous energy conditions, relying on batteries and cellular connectivity for data transmission. In this context, the remote telecontrol provided by PROMETEO not only guarantees continuity of acquisition, but also becomes an essential diagnostic tool.

The operational value of PROMETEO can be illustrated through several representative maintenance scenarios. In the first scenario, when the primary mains supply of a station fails, PROMETEO detects the loss of the 230 V supply and simultaneously monitors the response of the backup power system. The battery-voltage, current, and state-of-charge trends allow operators to follow the discharge process remotely. Analysis of the discharge curve provides an estimate of the effective battery capacity and helps determine whether the battery remains suitable for operation or should be replaced during the next field intervention. Considering the typical load of a mobile station—approximately 500 mA at 12 V—these measurements also allow the expected remaining operating time to be assessed before a complete station shutdown occurs.

A second maintenance scenario concerns stations powered by photovoltaic systems. PROMETEO records photovoltaic voltage and power, battery state of charge, and load current over time. The combined analysis of these parameters makes it possible to distinguish temporary reductions in solar production from inadequate system sizing or progressive battery degradation. Technical personnel can therefore evaluate remotely whether the available solar energy is sufficient for the installed load and whether additional photovoltaic panels and/or storage batteries are required.

A third scenario involves the differentiation between power-supply and communication failures. When a station stops transmitting scientific data, the simultaneous analysis of mains availability, battery voltage, equipment reachability, cellular or radio signal quality, and network-device status allows operators to determine whether the interruption is caused by an energy problem, a communication-link failure, or a malfunction of the acquisition equipment. This preliminary remote diagnosis enables the maintenance team to identify the instruments and replacement components required before travelling to the site, thereby avoiding exploratory or “blind” interventions.

PROMETEO can also support active energy management under critical operating conditions. If a progressive reduction in the available battery capacity is detected, remotely controlled relays can be used to disconnect non-essential devices and preserve the operation of the instrumentation required for scientific data acquisition and transmission. These capabilities are particularly valuable at remote sites, where access may require substantial travel time and complex logistical organization.

Thanks to the multiparametric approach, the system allows operators to rapidly distinguish between energy-related issues, such as battery discharge, and critical problems associated with communication infrastructures. This remote diagnostic capability drastically reduces the need for blind field interventions, which would otherwise be costly and logistically complex. Moreover, the immediate visibility of station health status increases the overall resilience of the infrastructure, making it possible to prioritize maintenance activities and prevent the loss of valuable scientific data caused by latent malfunctions.

## 4. Discussion

The operational deployment of the PROMETEO system within the monitoring infrastructures of the INGV—Osservatorio Vesuviano demonstrates the practical benefits of adopting an Internet of Things (IoT) paradigm for the management of distributed geophysical monitoring networks. In contrast with traditional monitoring approaches, which mainly focus on the acquisition and transmission of scientific measurements, the PROMETEO framework introduces a complementary infrastructure-level monitoring layer that allows continuous supervision of the technological components supporting data acquisition systems.

One of the main outcomes of this work is the demonstration that the integration of heterogeneous protocols within a unified supervisory architecture can significantly improve the operational visibility of complex monitoring infrastructures. The combined use of MQTT, SNMP, and MODBUS enables the system to collect and correlate information from devices with different communication paradigms and operational roles. MQTT provides an efficient and lightweight transport mechanism suitable for low-bandwidth environments typical of remote monitoring stations. SNMP [16] allows periodic interrogation of network infrastructure devices and communication equipment, while MODBUS enables direct acquisition of electrical and industrial parameters with high temporal resolution. The coexistence of these protocols within a single supervisory platform allows the PROMETEO system to represent multiple layers of infrastructure status, ranging from energy sustainability to network connectivity and environmental conditions.

The results highlight the importance of multiparametric monitoring in improving diagnostic capability within distributed monitoring systems. The simultaneous observation of electrical parameters, environmental variables, and communication indicators enables operators to rapidly identify the root causes of anomalies affecting monitoring stations. For example, the correlation between battery voltage trends, current consumption, and communication signal strength allows differentiation between energy-related failures and network connectivity issues. This capability significantly reduces diagnostic uncertainty and improves response time when infrastructure anomalies occur.

Another relevant aspect concerns the use of the Home Assistant platform as the central supervisory hub. Although originally designed for smart home automation, Home Assistant has demonstrated considerable flexibility in the context of scientific infrastructure monitoring. Its modular architecture, extensive protocol support, and integration ecosystem allow the platform to handle heterogeneous data sources and monitoring tasks. Within the PROMETEO framework, Home Assistant enables real-time visualization through customized dashboards, historical storage of diagnostic parameters, and the implementation of automated alert mechanisms. This approach also offers advantages in terms of cost-effectiveness and scalability, as the platform is open-source and can be easily extended to support additional devices and monitoring capabilities.

The contribution of PROMETEO is not intended to claim the novelty of remote monitoring itself, nor of the individual technologies adopted. Rather, its specific contribution lies in the integration, within a single supervisory environment, of heterogeneous operational information related to different technological layers of the monitoring infrastructure, including power supply and energy storage, network connectivity, communication equipment, and the operational status of field devices through the coordinated use of different standard protocols. This approach complements traditional scientific-data acquisition with an infrastructure-level supervision layer specifically oriented toward routine observatory operations.

From an operational perspective, the system contributes to improving the resilience of the monitoring network. By enabling early identification of degradation processes—such as battery deterioration, abnormal power consumption, or unstable network connectivity—PROMETEO supports preventive and predictive maintenance strategies. This reduces the need for reactive interventions and helps optimize the allocation of technical resources, particularly in the management of mobile seismic stations that may require complex logistics for field maintenance.

An important advantage of intelligent software hubs such as Home Assistant is the possibility of implementing automated control strategies within the platform. These automations can be defined through YAML configurations and/or Python scripts and can autonomously execute predefined actions according to the observed operational state of the monitored system. For example, an automation can reduce the overall power consumption of a station when a critical energy condition is detected by selectively switching off less essential equipment through dedicated relays controlled by MQTT commands, thereby preserving battery capacity and maintaining the operation of the most critical instrumentation until the external power supply is restored. Future developments may include the integration of data analytics and machine learning techniques for anomaly detection and predictive modeling based on historical infrastructure data.

Furthermore, the architecture could be extended toward greater interoperability with other monitoring platforms and data infrastructures used in geophysical observatories. The adoption of standardized data models and interoperability frameworks would facilitate the integration of PROMETEO with broader scientific data management systems.

Overall, the results of this study indicate that the integration of IoT technologies, multiprotocol communication architectures, and open-source supervisory platforms represents a promising strategy for improving the operational management of complex geophysical monitoring infrastructures. The experience gained with the PROMETEO system provides a practical example of how infrastructure-level monitoring can enhance the reliability and resilience of distributed sensor networks operating in challenging environmental contexts.

## 5. Conclusions

PROMETEO follows a hub-and-spoke architecture in which the software hub represents the core of the entire system, while the monitored devices are distributed across remote field sites. The central hub runs on a dedicated server located in the institutional Data Processing Center (CED).

The system centralizes heterogeneous data, stores them in time-series databases, supports real-time analysis and correlation, enables automations for predictive analysis of system behavior, and distributes information and alerts through multiple platforms, including e-mail, Telegram, and application notifications. The architecture is extensible toward additional standard or customizable protocols, supported commercial devices, web-based visualization for authorized users, and potential future integration with Artificial Intelligence modules.

PROMETEO enables rapid fault diagnosis through the analysis of remotely acquired data, remote control of field devices, and the implementation of automated operating strategies in response to failures occurring at monitoring sites.

Future developments include the progressive integration of an increasing number and variety of devices and the interaction of the PROMETEO environment with Artificial Intelligence modules.

## Figures and Tables

**Table 1:** Comparison of the main features of selected home automation platforms.

Feature	Home Assistant	openHAB	Domoticz	Hubitat Elevation
**Software Type**	Open Source	Open Source	Open Source	Proprietary paid hardware
**Architecture**	Local and/or Cloud	Local and/or Cloud	Local	Local and/or Cloud
**Ease of Use**	Medium	Medium-High	Medium	Medium-Low
**Device Compatibility**	Excellent	Very good	Good	Very good (focused on Zigbee and Z-Wave)
**Hardware Requirements**	Raspberry Pi, Linux PC, etc.	Raspberry Pi, Linux PC, etc.	Lightweight hardware (including older PCs)	Dedicated proprietary hub
**Automation Language**	Visual editor, YAML, Python v3.14.6	Visual rules, Java, JavaScript	Blockly, Lua, Python v3.14.6	Rule Machine (advanced visual tool)
**Community and Support**	Huge and highly active	Large and well structured	Moderate	Active (via forums)

**Table 2:** Main device classes monitored by PROMETEO, with models, monitored parameters, communication protocols, and polling intervals.

Device Type	Model	Monitored Parameters	Communication Protocol	Polling Interval
**Data acquisition system**	Guralp Affinity	Seismometer mass position, supply voltage, visible GPS satellites, etc.	HTTP	Every 5 min
**Solar power controller**	Victron Cerbo GX	Voltage, current, state of charge (SoC), PV voltage, power, etc.	MQTT	Every 5 min
**Mains power detection/control system**	Shelly Pro 1PM	230 V mains availability, ON/OFF control	MQTT	Every 5 min and/or client-triggered
**Electrical energy measurement system**	Finder energy meter	Mains availability, 230 V energy consumption, etc.	MODBUS	Every 5 min
**Communication system**	MikroTik Wi-Fi radio lhg xl	RSSI, bit rate, link signal quality, etc.	SNMP	Every 5 min

## Data Availability

Data are available from the authors upon reasonable request.

## Supplementary Materials

The following supporting information can be downloaded at: https://www.mdpi.com/article/10.3390/s26185914/s1, Supplementary File S1: Example of JSON-formatted MQTT payload used for remote station telemetry; Supplementary File S2: Example of YAML configuration for Modbus/TCP electrical meter monitoring.
