# Supplementary material for: PROMETEO: Infrastructure Remote-Control and Geophysical Monitoring System of the INGV Osservatorio Vesuviano
Source: Sensors (Basel). 2026 Sep 18;26(18):5914. doi: 10.3390/s26185914 (PMC13611512; doi:10.3390/s26185914)
Supplement: Supplementary file 1 [file sensors-26-05914-s001.zip › sensors-4431217-supplementary.pdf]

MDPI - Sensors

Special Issue "Next-Generation Geophysical Sensing"

Supplementary material for

"PROMETEO: Infrastructure Remote-Control and Geophysical Monitoring System of the INGV Osservatorio Vesuviano"

Aldo Benincasa[1], Antonio Caputo[1], Francesco Liguoro[1], Giovanni Scarpato[1], Massimo Orazi[4], Roberto Manzo[1*]

[1]Istituto Nazionale di Geofisica e Vulcanologia, Sezione di Napoli, Osservatorio Vesuviano

*Correspondence: roberto.manzo@ingv.it

Contents of this file:

S1: Example of MQTT telemetry payload used for remote station monitoring.


```json
{
  "timestamp": "2023-10-27T14:30:00Z",
  "power_supply": {
    "battery_voltage_V": 12.8,
    "load_current_A": 0.45,
    "status": "discharging"
  },
  "environment": {
    "cabinet_temperature_C": 24.5
  },
  "connectivity": {
    "rssi_dBm": -65,
    "uptime_s": 3600
  }
}
```

S2: Example of electrical meter configuration.


```yaml
- name: CAAM Contatore
  type: tcp
  host: 10.212.31.3
  port: 502

  sensors:
    - name: CAAM Contatore U1 RAW
      slave: 1
      address: 107
      input_type: input
      data_type: custom
      structure: ">2h"
      scan_interval: 5

    - name: CAAM Contatore I1 RAW
      slave: 1
      address: 126
      input_type: input
      data_type: custom
      structure: ">2h"
      scan_interval: 5
```
